# The characterisation of sexual behaviour in Chinese male university students who have sex with other men: A cross-sectional study

**DOI:** 10.1186/1471-2458-8-250

**Published:** 2008-07-22

**Authors:** Liming Cong, Masako Ono-Kihara, Guozhang Xu, Qiaoqin Ma, Xiaohong Pan, Dandan Zhang, Takayuki Homma, Masahiro Kihara

**Affiliations:** 1 Center for Disease Control and Prevention of Zhejiang Province, Hangzhou, PR China; 2 Department of Global Health and Socio-epidemiology, Kyoto University School of Public Health, Kyoto, Japan; 3 Center for Disease Control and Prevention of Ningbo Municipality, Ningbo, PR China; 4 Department of Drug Management & Policy, Faculty of Pharmacy, Kanazawa University Institute of Medical, Pharmaceutical and Health Sciences, Kanazawa city, Japan

## Abstract

**Background:**

The risks for Chinese male university students who have sex with other men (MSM) have not been compared with those for non-MSM students. This information is important for the development of targeted HIV prevention programmes for this population.

**Methods:**

Sexually active MSM and non-MSM students were compared for demographic characteristics, sexual behaviour, and related psychosocial variables using bivariate analyses. The data were a subset drawn from a large-scale cross-sectional questionnaire survey of sexually active male students conducted at two universities in a large city in Zhejiang Province, China, in 2003.

**Results:**

Of 1824 sexually active male students, 68 (3.7%) reported having had sex with a man at least once; 33.8% of these 68 men had also had female partners. Compared with non-MSM students, MSM students were 3–6.5 times more likely to have had sexual encounters with casual or commercial sex partners and were three times less likely to have protected sex in the past year or during their lifetime. They were three to five times more likely to have had multiple partners and 15 times more likely to have had a sexually transmitted disease (STD). In addition, the MSM students knew half as much about HIV and had less condom-decision than did non-MSM students and were two times more accepting of commercial sex. However, the MSM students were twice as aware of the risks for HIV infection.

**Conclusion:**

MSM composed 3–4% of the male sexually active university student population studied and was found to be at greater risk than non-MSM students for STD/HIV infection. There is an urgent need for STD/HIV programmes in university health services that take into consideration the sexuality and psychosocial issues of MSM students.

## Background

China, the most populous nation in the world, is experiencing an increase in HIV/AIDS. National sentinel surveillance data indicate that from 1996 or 1997 to 2004, HIV prevalence rose from 1.95 to 6.48% in injection drug users, from 0.02 to 0.93% in female sex workers, and from 0 to 0.26% in pregnant women [1,2]. Although injection drug use and sexual contact are currently the predominant modes of HIV transmission, sexual transmission is on the rise and is estimated to have increased from 30% in 2003 to 44% in 2005 and 57% in 2007 [1-4].

Recent reports indicate that there is an HIV epidemic among men who have sex with men (MSM) in Asian countries. MSM constitute 15–45% of all HIV infections in Taiwan and Hong Kong [5,6], and studies conducted in Cambodia and Thailand indicate that the prevalence of HIV in MSM is 15 and 17%, respectively [7,8]. Since the first MSM sexually transmitted HIV case was detected in Beijing in 1989 [9], several studies have shown that an HIV epidemic is emerging in Chinese MSM. A serial survey conducted between 1998 and 2001 found that the prevalence of HIV in MSM was 2.5–17.1% [10]. In Shenzhen, the prevalence of HIV in MSM increased from 0.9% in 2002 to 2.7% in 2005 [11], and in Beijing it increased from 0.5% in 2004 to 5% in 2006 [12]. Estimates by China's Health Ministry and the Joint United Nations Programme on HIV/AIDS indicate that MSM accounted for 7% of HIV infections in China through 2005 [2] and for 12.2% of the cases in 2007 [4].

Several studies performed in China suggest that MSM are vulnerable to sexually transmitted disease (STD) and HIV [13-17] because they have unprotected sexual intercourse. For example, only 23–31% of MSM reported always using a condom during insertive anal sex, 28–34% during receptive anal sex in Jinan and Nanchang during the previous 6 months [16,17], and 32% reported always using a condom during anal sex in a six-city study in 2004 [15]. However, the interpretation of these data is limited by the fact that most of the data were derived from convenience samples collected in gay bars, bathhouses, parks, on the Internet, and from voluntary counselling and testing centres without non-MSM controls. They do not represent the entire MSM population and it is difficult to evaluate if and how their sexual behaviour is more at risk compared to non-MSM population.

In 2003, we conducted a large-scale survey of university students with a response rate of 76.5%. This provided us with the opportunity to determine the proportion of MSM in sexually active student population and to characterise the history, practice, and psychosocial aspects of their sexual behaviour and compare it with that of non-MSM students.

## Methods

### Setting and participants

We obtained data from a large cross-sectional survey administered in 2003 with a sample of 22,493 students (response rate of 76.5%) from two universities located in a large Chinese coastal city in Zhejiang province [18]. The city contains only two universities. Because both universities participated in the study, opportunity was given to sample entire university student population in the city. Of the 22,493 participants, 17.6% (1981 of 11,255) of the males and 8.6% (963 of 11,238) of the females were sexually active.

### Data collection

The questionnaire was developed based on literature reviews, qualitative research, a pilot study, and a test-retest reliability study, as described previously [18]. The final version of the questionnaire included questions on: sociodemographic characteristics (15 items); knowledge about HIV, STD, and contraception (37 items); attitude towards an HIV-positive person and the perception of risk for HIV (5 items); exposure to sexual information and experience in having a boyfriend or girlfriend (7 items); sexual behaviours during the first, lifetime, past year, and most recent sexual contact (31 items); ability to reject unwilling sex (1 item); comfort with obtaining and using condoms (3 items); attitude towards sex (13 items); self-evaluation (2 items); and comments on sex education (4 items). The variables included in this paper were selected among these questions.

All students in Years 1–4 at the two universities were asked to fill out the self-administered survey. Trained staff distributed and collected the questionnaire. The local education board, the two universities involved, and the institutional review body of the Zhejiang Provincial Centre for Disease Prevention and Control reviewed the research protocol and instrument, and approved its use. As a process of informed consent, all participants were informed of the study's purpose, and invited to the study being explained how, when and where the survey was to be conducted. They are also told that non-participation would cause no disadvantage to them, and the survey was anonymous and the data was to be presented only in an aggregated manner, therefore the participants' privacy and confidentiality would be firmly protected. All these policies were also printed in the front cover of the questionnaire.

### Statistical analysis

In addition to the sociodemographic and sexual behaviour variables, our study created two psychosocial scales for statistical analysis: HIV knowledge and condom-decision. The HIV-knowledge scale consisted of 10 statements with a Cronbach's alpha coefficient of .667. The 10 statements required a response of "correct," "incorrect," or "do not know," including HIV can be transmitted through (1) sex without protection, (2) syringe or needle sharing, (3) mother-to-child contact, (4) blood transfusion, (5) working together; (6) STD infection increases HIV transmission; condom use can prevent (7) HIV and (8) other STDs; (9) China has a rapidly growing HIV epidemic; and (10) sexual transmission of HIV is a major factor in the HIV epidemic in China. The condom-decision scale consisted of three statements that were designed to determine whether the students make the decision to use a condom prior to sex, if they use a condom when having sex with a partner who is important to them, and whether they are comfortable buying a condom for themselves or their partner. The Cronbach's alpha coefficient for this scale was .514. The condom-decision scales were measured using a 3- to 5-point Likert-type scale, respectively, ranging from positive to negative responses. Participants were categorised into low- and high-score groups based on the median distribution of the total scores for each scale.

The data were analysed using SPSS for Windows (version 12.01; SPSS Inc., Chicago, IL). A bivariate analysis was performed to identify variables that were significantly associated with MSM status and was expressed as an odds ratio (OR) with a corresponding 95% confidence interval (CI). Statistical significance was set at *p *< 0.05.

## Results

Sexually active male students who had had oral, anal, or vaginal sex and reported the gender of their sexual partner were included in the study. The sample size was 1824 students. Of these, students who reported that their partners were male or both male and female were identified as MSM; those whose sexual partners were exclusively female were identified as heterosexual, or non-MSM.

Of all sexually active male students, 68 (3.7%) reported having had sex with men, of which 23 (33.8%) reported also having had sex with women. There was no difference between MSM and non-MSM students in demographic or lifestyle variables, including the frequency of dancing, cigarette smoking, and karaoke singing (Table 1). However, the MSM group drank less frequency than did the non-MSM group (*p *= 0.041).

**Table 1:** Sociodemographic characteristics of the respondents

	**MSM (n = 68)**		**Non-MSM (n = 1756)**	
		
	n	%^a^	n	%^a^
University				
A	42	61.8	981	55.9
B	26	38.2	775	44.1
Age				
≤ 19	7	10.3	249	14.2
> 19	58	85.3	1498	85.3
Year of study				
I	10	14.7	399	22.7
II	21	30.9	480	27.3
III	28	41.2	617	35.1
IV	9	13.2	260	14.8
Hometown area				
Rural	17	25.0	417	23.7
Town/city	51	75.0	1333	75.9
family's economic status				
Rich	13	19.1	217	12.4
Between	48	70.6	1418	80.8
Poor	7	10.3	117	6.7

^a ^The percentage of respondents may not add up to 100% due to non-response for some items.

### Sexual behaviours

There was no difference between groups in the age at which they first had sex. The first sexual encounter for MSM students was more likely to have been with a casual partner (25.0 vs. 10.9%; OR: 3.15) or commercial partner (8.8 vs. 1.9%; OR: 6.24), and they were more likely than non-MSM students to experience unwilling sex (11.8 vs. 2.4%; OR: 6.17) or coerced sex (7.4 vs. 0.3%; OR: 27.0; Table 2). MSM students had a tendency not to use a condom during the first sexual encounter, but it was not statistically significant (OR: 1.78, 95% CI: 0.97–3.29). MSM students had significantly more partners during their lifetime than did non-MSM students (55.9 vs. 33.3%; OR: 5.32), but 61.8% of MSM students never or rarely used condoms compared to 34.9% of non-MSM students (OR: 3.29). MSM students were significantly more likely to have had a history of STD than were non-MSM students, at 12 and 1%, respectively (OR: 15.25).

**Table 2:** Sexual behaviours of men who have sex with men (MSM) versus heterosexual men (non-MSM) among sexually active male students, China

	**MSM (n = 68)**		**Non-MSM (n = 1756)**		**Crude OR(95%CI)**^b^	***P Value***
				
	n	%^a^	n	%^a^		
**First sex**						
Partner type in first sex						
Regular	43	63.2	1521	86.6	1	
Casual	17	25.0	191	10.9	3.15(1.76–5.63)	0.000
Commercial	6	8.8	34	1.9	6.24(2.49–15.65)	0.000
Condom use in first sex						
Yes	13	19.1	530	30.2	1	
No/not sure	53	77.9	1215	69.2	1.78(0.97–3.29)	0.067
Consent at first sex						
Willing	52	76.5	1685	96.0	1.0	
Unwilling	8	11.8	42	2.4	6.17(2.76–13.80)	0.000
Being coerced	5	7.4	6	0.3	27.00(7.98–91.32)	0.000
						
**Sex in lifetime**						
Partner number lifetime						
1	13	19.1	1062	60.5	1	
≥ 2	38	55.9	584	33.3	5.32(2.81–10.06)	0.000
Condom use life time						
Always/often/sometimes	23	33.8	1113	63.4	1	
Rarely/Never	42	61.8	612	34.9	3.29(1.96–5.53)	0.000
STD diagnosed lifetime						
No	51	75.0	1653	94.1	1	
Yes	8	11.8	17	1.0	15.25(6.29–36.97)	0.000
						
**Sex in the last year**						
Partner type in the last year^c^						
Regular only	27	55.1	1139	84.1	1	
Ever casual^d^	14	28.6	150	11.1	3.94(2.02–7.68)	0.000
Ever commercial^e^	6	12.2	40	3.0	6.33(2.47–16.19)	0.000
Partner number in the last year^c^						
1	24	49.0	1040	76.8	1	
≥ 2	18	36.7	279	20.6	2.80(1.50–5.22)	0.001
Condom use in the last year^c^						
Always/often/sometimes	18	36.7	820	60.8	1.0	
Rarely/Never	30	61.2	503	37.3	2.72(1.50–4.93)	0.000

^a ^The percentage of respondents may not add up to 100% due to non-response for some items.

^b ^OR, odds ratio; CI, confidence interval.

^C ^Male students who were sexually active in the last year, n = 49 for MSM, and 1354 for non-MSM.

^d ^Including those who had had a casual partner at least and those who had had both casual and regular partners.

^e ^Including those who had had a commercial partner at least.

Of all of the participants in each group, 49 (72.1%) MSM and 1354 (77.1%) non-MSM students had been sexually active during the past 12 months. During this period, MSM students were more likely to have had at least one casual partner (28.6 vs. 11.1%; OR: 3.94) and/or to have been with at least one commercial partner (12.2 vs. 3.0%; OR: 6.33), and were more likely to have had multiple partners (36.7 vs. 20.6%; OR: 2.80). MSM students were less likely to use a condom, with 61.2% reporting that they never or rarely used a condom compared to 37.3% in the non-MSM group (OR: 2.72).

### Psychosocial characteristics

Awareness of the risk for contracting HIV was low in both groups. However, MSM students were significantly more aware of the risks than were non-MSM students (32.4 vs. 20.1%; OR: 1.93; Table 3). MSM students were significantly more likely than non-MSM students to approve of commercial sex (50.0 vs. 33.8%; OR: 2.38), and they knew less about HIV (45.6 vs. 28.1%; OR: 2.28) and had lower condom-decision (55.9 vs. 41.2%; OR: 2.04).

**Table 3:** Psychosocial characteristics of men who have sex with men (MSM) versus heterosexual men (non-MSM) among sexually active male students, China

	**MSM (n = 68)**		**Non-MSM (n = 1756)**		**Crude OR(95%CI) **^b^	***P Value***
				
	n	% ^a^	n	% ^a^		
Risk awareness for HIV						
No/low possible	43	63.2	1333	75.9	1	
Some-high possible/not sure	22	32.4	353	20.1	1.93(1.14–3.27)	0.014
Approve of commercial sex						
Disapprove/not sure	25	36.8	1038	59.1	1	
Approve	34	50.0	593	33.8	2.38(1.41–4.03)	0.001
HIV knowledge scale						
High(9–10)	34	50.0	1231	70.1	1	
Low(0–8)	31	45.6	493	28.1	2.28(1.38–3.75)	0.001
Condom–decision scale						
High(10–12)	23	33.8	895	51.0	1	
Low(3–9)	38	55.9	724	41.2	2.04(1.21–3.46)	0.008

^a^The percentage of respondents may not add up to 100% due to non-response for some items.

^b ^OR, odds ratio; CI, confidence interval.

## Discussion

This is the first study in China to report the proportion of MSM in a male sexually active university student population and to characterise MSM psychosocial factors and sexual behaviour and compare them to those of non-MSM students. Of the sexually active male university students studied, 3.7% had had sex with a man at least once. Previous studies of 15–49-year-old men in 10 Western countries found that 0.9–13.4% (median: 5.0%) had had sex with a man [19]. Our lower estimate might be due to underreporting by respondents for fear of stigmatisation or to the lower age range of our survey. However, our findings on sexual behaviours are consistent with other recent surveys in China. One-third of MSM reported having had sex with both men and women; this agrees with previous research that indicates that Chinese MSM are commonly bisexual [13-17]; thus the reported MSM data in this paper are on mixed population of MSM having sex only with men and those having sex with both men and women. Recent reports on condom use indicated that in the previous 6 months only 24–27% of MSM always used a condom with a female partner and 23–34% always used a condom during anal sex with a male partner [15-17]; we found that in the previous 12 months, only 7.7% of the MSM respondents always used a condom.

The MSM participants reported frequent encounters with sexual partners, especially with casual or commercial sex partners. This finding agrees with previous studies in China [16,20]. Our finding that 12% of MSM students had had at least one STD is similar to data reported in previous studies of Chinese MSM [13-16,20,21]. Our data indicate that MSM students studied engaged in the same level of risky sexual behaviour shown by MSM in previous studies, suggesting that they are likely to become part of the HIV epidemic that is emerging in MSM in China.

We quantified the increased sexual risk of MSM students compared to non-MSM students. MSM students studied had 3–6.5 times more sexual encounters with casual or commercial sex partners, were three times less likely to use a condom, were three to five times more likely to have multiple partners, and were 15 times more likely to contract an STD during the previous year or during their lifetime. In addition, MSM students studied were half as informed about HIV and had less condom-decision than did non-MSM students, and were two times more accepting of commercial sex. However, MSM students were twice as aware of the risk for HIV infection. Reason of the lower HIV-related knowledge but higher awareness for the risk of HIV infection among MSM university students studied is unclear but it may be due to the confounding of demographic variables because association of lower HIV-related knowledge but not risk awareness for HIV with MSM status disappeared in multivariate analysis (data not shown), or it may be that MSM students studied had minimum knowledge that unprotected sex may place them at risk for HIV infection but lack broader knowledge on HIV because of their limited access to HIV-related information.

These results clearly demonstrate that MSM university students studied are at particular sexual risk. In view of the fact that STD, which increases susceptibility to HIV infection, is already prevalent in MSM students, prevention, STD/HIV testing, treatment, and counselling and support programmes for MSM university students must be a high priority for university health services. Homosexuality is highly stigmatised in Chinese society [22,23], and MSM students face complex psychosocial issues regarding sex and STD. This is indicated by the high incidence of coerced sex, which is associated with unprotected sex, multiple partnerships [24-26], limited understanding of HIV, and low condom-decision. However, MSM students studied had a liberal attitude towards sex and a high awareness of the risk for HIV infection. University STD/HIV prevention programmes that pay particular attention to MSM students' sexuality and their complex psychosocial situation need to be developed.

Our study has some limitations. First, it was restricted to two universities in one city, and the sample may not be representative of MSM university students across China. Second, the cross-sectional nature of the study makes it difficult to determine causal relationships. Third, the data may be biased because the questionnaire was self-reported; there was a relatively large amount of missing data for some variables, and 23.5% of the students did not respond to the survey. Fourth, the questions were not designed specifically for MSM students, and important information such as roles in sex (insertive or receptive), the use of cruising spots, and various psychological aspects were missing or incomplete. Thus, the data may not fully describe the sexual and psychosocial characteristics of MSM students. Further studies with larger sample size are clearly needed to make deep insight into the psychosocial needs, characteristics of MSM student's sexual behaviour and its context, and to explore the differences between MSM students with different level of risk taking behaviour. Such information may provide the basis for culturally appropriate STD/HIV prevention programmes for this subpopulation in China.

## Conclusion

MSM practices were reported by 3.7% of male students from two universities in one coastal city in China. The sexual practices of these students was as risky as that reported by MSM in other studies and much more risky than that reported by non-MSM university students. In fact, > 10% of MSM students experienced STD infection. In view of the emerging HIV epidemic in MSM in China, there is an urgent need for STD/HIV programmes in university health services that take into consideration the issues of MSM students.

## Abbreviations

MSM: Men Who Have Sex with Men; STD: Sexually Transmitted Disease; HIV: Human Immunodeficiency Virus; AIDS: Acquired Immune Deficiency Syndrome.

## Competing interests

The authors declare that they have no competing interests.

## Authors' contributions

All authors contributed to the design of this research. LC and QM performed the statistical analysis and drafted the manuscript; LC and GX coordinated the study in field; QM, XP, and DZ played a major role in the field survey; TH helped analyze the data; MO–K and MK supervised the research, statistical analysis and revised the manuscript. All the authors of the manuscript have read and agreed to its content.

## Pre-publication history

The pre-publication history for this paper can be accessed here:


